# Psychometric properties of the Arabic Occupational Balance Questionnaire (OBQ11-A)

**DOI:** 10.1080/07853890.2024.2346945

**Published:** 2024-04-27

**Authors:** Brightlin Nithis Dhas, Samah Ahmad Abd Alfattah Abd Alhadi, Ghaith Mohammad Rizk Dhadl Al That, Sultan Salim Hammam Al Abdulla

**Affiliations:** a Faculty of Health, Education & Society, University of Northampton, United Kingdom; b Occupational Therapy Department, Hamad Medical Corporation, Doha, Qatar

**Keywords:** Arabic, occupational balance, rehabilitation, reliability, validity

## Abstract

**Background:**

Occupational balance (OB) is a desirable outcome of rehabilitation because it is related to various health indices. The Occupational Balance Questionnaire (OBQ) is a self-report measure of occupational balance.

**Aims/Objectives:**

To examine the test-retest reliability, participant-level content validity, construct validity, internal consistency, and convergent validity of an Arabic occupational balance questionnaire (OBQ11-A).

**Materials and Methods:**

A total of 103 participants were recruited through convenience sampling. Test-retest reliability was examined using intraclass correlation coefficients, participant-level content validity using percentage of agreement in survey questions, construct validity using factor analysis, internal consistency using Cronbach’s alpha, and convergent validity by examining associations with quality-of-life.

**Results:**

Intraclass Correlation Coefficient values for the total OBQ11-A scores and individual items were greater than 0.7 between the test and retest. The majority of respondents endorsed the relevance, comprehensiveness, and comprehensibility of the OBQ11-A. Cronbach’s alpha for the OBQ11-A total score was 0.899. Nine of the 11 OBQ11-A items had factor loadings greater than 0.7. Moderate associations were found between the total OBQ11-A scores and physical health (*n* = 101; ρ = .52, *p* < .001), psychological health (*n* = 101; ρ = .49, *p* < .001), social relationships (*n* = 101; ρ = .36, *p* < .001), and environmental domains (*n* = 101; ρ = .57, *p* < .001) of the quality-of-life measure.

**Conclusions:**

OBQ11-A demonstrates acceptable psychometric properties for research purposes.

## KEY MESSAGES

Occupational balance, referring to an individual’s self-perception of having the right amount and variety of occupations in his/her occupational pattern, is associated with various health indicators and could serve as an additional outcome of rehabilitation.

The Arabic version of the Occupational Balance Questionnaire (OBQ11-A) was found to have acceptable validity and reliability for use among Arabic-speaking populations.

## Introduction

Occupational balance (OB) refers to an individual’s self-perception of having the right amount and variety of occupations in his/her occupational pattern [1]. OB is related to a number of health indicators, such as quality of life [2–5], psychological wellbeing [6], perceived physical and mental health [7, 8], sleep quality [9], reduced stress levels [8, 10, 11] life satisfaction [5, 12, 13], and family quality of life [14]. Measuring the level of OB and addressing OB issues among clients are important for occupational therapists working in rehabilitation settings [15]. OB can be a useful outcome measure in rehabilitation, as an imbalance in daily occupation is found to negatively affect health.

In occupational therapy, several approaches have been proposed to measure OB [16–20]. The Occupational Balance Questionnaire (OBQ/OBQ11) is a self-report tool developed to measure an individual’s perception of OB [20, 21]. It was originally formulated in Swedish and later translated into English by Yu et al. (2018) [8]. The instrument was developed based on the principle that OB is subjective (perceived by the individual), individualistic (different occupations could have different values for the individual), and satisfaction with time spent on different occupations rather than actual time use [20]. The OBQ has 13 items rated on a six-point ordinal rating scale, with higher scores suggesting higher occupational balance [20]. Håkansson et al. (2020) revised it by removing two items based on Rasch analysis and reducing the response options to a four-point rating scale, resulting in an 11-item measure (OBQ11), with total scores ranging from 0 to 33 [21]. The OBQ is a useful tool to measure OB in different population groups and is being used extensively in epidemiological research to explore the various factors associated with OB and the implications of having low levels OB among individuals [13].

The items for the OBQ were derived from perceptions of balance in everyday life among different population groups and a concept analysis of OB-related articles in different contexts [20, 21]. The OBQ has been translated and validated in Spanish [5, 13] and the OBQ-11 in Arabic [22], French [23], Norwegian [24, 25], Polish [6], and Turkish [26]. Validation of translated versions of measurement instruments is an essential methodological requirement to use the instrument in research and to endorse its use in clinical practice. In a preliminary validity study of the Arabic version (OBQ11-A), Dhas et al. (2022) found evidence for content validity (among Arabic-speaking occupational therapists), internal consistency, and a positive association with family quality of life [22]. In order to establish the content validity of a questionnaire, Terwee et al. (2018) suggested that both professionals and participants need to be probed about its relevance specific to population being studied and their contexts; its comprehensiveness in terms of inclusion of all key aspects of the construct; and its comprehensibility whether all items are understood by participants as intended [27]. In the preliminary validity study of OBQ11-A, construct validity was established only from professionals and not participants. Moreover, the test–retest reliability of OBQ11-A was not examined. Therefore, this study was conducted to fill these gaps in the preliminary validation study with the primary objective of examining the test–retest reliability of OBQ11-A and the participant-level content validity. The secondary objectives are to examine its construct validity, internal consistency, and convergent validity in a large general population sample.

## Materials and methods

This study used a cross-sectional design. Arabic-speaking staff and visitors from two major hospitals in the Hamad Medical Corporation were recruited through convenience sampling between February and August 2022. Those who self-identified themselves to have any type of disabilities, those who could not read and write in Arabic, and those who did not provide consent were excluded. Potential participants were approached through email and/or face-to-face at their offices (for staff) and waiting areas (visitors), and the research was explained. Two visits were required for each participant. During the first visit, informed consent procedures were completed, followed by the filling of the data collection instruments by the participants. All participants signed a written informed consent. A second visit was scheduled 7 to 10 days after the first visit [26], during which OBQ11-A was completed for the second time by the participants along with the content validity questions. A sample size of seven times the number of questions in the questionnaire was advised for studies examining construct validity [28]. The OBQ11-A has 11 items, which required a sample of 77. However, a sample size of 100 was required for studies examining the internal consistency, and test–retest reliability of self-reported outcome measures [28, 29]. Therefore, a sample of 120 participants was recruited, considering the potential incomplete datasets. A total of 127 people were approached to meet the specified sample of 120 (five did not give consent and two identified themselves to have disability), and 103 returned all the study questionnaires. Ethical approval for the study was provided by the institutional review board of the Hamad Medical Corporation (MRC-01-21-708).

### Instruments

#### Demographic questionnaire

An investigator-developed demographic questionnaire was used to collect information on participant characteristics including gender, nationality, education, marital status, employment, and number of children.

#### Arabic occupational balance questionnaire (OBQ11-A)

The OBQ11-A is a self-reported measure to assess an individual’s perception of their OB in Arabic [20, 21]. The OBQ11-A has 11 items measured on a four-point rating scale (0 = completely disagree and 3 = completely agree), generating total scores ranging from 0 to 33. The OBQ11-A was translated from the English version of OBQ in three stages including forward and back translation and expert committee review in the first stage, field testing and cognitive interviews with 10 occupational therapists and 7 volunteers for content validity in the second stage, and preliminary validation among 67 Arabic speaking parents in the third stage [22]. During the preliminary validation stage, OBQ11-A was found to have good internal consistency (Cronbach’s alpha, 0.864), acceptable factor loadings for all items in exploratory factor analysis, and a positive statistically significant association with family quality of life scores (*r* = 0.561, *p* < .001), supporting its convergent validity [22].

The original occupational balance questionnaire (OBQ) that was developed in Swedish had 13 items and was reported to have good internal consistency (Cronbach alpha, 0.94) and test–retest reliability (Spearman’s Rho, 0.93) [20]. Later, two items from the OBQ suggesting multidimensionality were removed based on Rasch analysis resulting in the formulation of the revised 11-item occupational balance questionnaire (OBQ11) [21]. The revised OBQ11 exhibited response categories that worked properly, good reliability (Pearson Separation Index of 0.92), model fit, and measurement invariance across age and gender groups [21]. The English version demonstrated acceptable test-retest reliability (Spearman’s Rho, 0.74) and good convergent validity [8].

#### Content validity survey

To verify content validity, a survey was formulated to rate the relevance, comprehensiveness, and comprehensibility of OBQ11-A [27] and administered to participants during the second administration of OBQ11-A. The following seven questions adapted from Alnahhal and May (2012) [30] were included in the survey: 1. The questions were clear and easy; 2. The questions covered all aspects of occupational balance (balance in everyday activities); 3. Would you recommend this questionnaire to another volunteer? 4. It took a lot of time and effort to complete the questionnaire; 5. The questions appeared to encourage specific answers; 6. Did you find it difficult to answer any of the questions; 7. The questionnaire lack important questions regarding occupational balance. Responses were recorded on a 5-point Likert scale (strongly agree, agree, undecided, disagree, strongly disagree).

#### World health organization quality of life questionnaire (WHOQOL-BREF)

The WHOQOL-BREF is a shorter version of the WHO quality of life assessment instrument [31]. It consists of 26 items grouped into four domains: physical health (seven items), psychological health (six items), social relationships (three items), and environment (eight items). In addition, there were two single items that measured the overall perception of quality of life and overall perception of health. The WHOQOL-BREF is scored on a 5-point Likert scale. Different anchors, including very poor to very good, very dissatisfied to very satisfied, not at all to an extreme amount, and never to always, were used depending on the type of questions. Mean scores for each domain were multiplied by 4 and subsequently transformed to a 0–100 scale in order to make them comparable with the full 100-item version of the WHOQOL-100 [31]. In previous studies, the coefficient alpha of the Arabic WHOQOL-BREF scales ranged from 0.69 to 0.93, indicating acceptable internal consistency, and test-retest reliability was significant (ICC = 0.95) [32]. The Arabic WHOQOL-BREF was used to examine the construct validity of the OBQ11-A.

### Statistical analysis

Statistical analyses were performed using SPSS version 27 and SPSS AMOS software. The test-retest reliability of the OBQ11-A between the first and second evaluations was assessed using the intraclass correlation coefficient (ICC). For a research instrument, a reliability of 0.70, was considered acceptable [27]. ICC values were calculated for each item and the total OBQ11-A score. The percentage of agreement on the validity question was used to demonstrate participant-level content validity. Construct validity was determined using an explanatory factor analysis. The Kaiser–Meyer–Olkin (KMO) value and Bartlett’s test of sphericity were used to examine the suitability of the factor analysis. Cronbach’s alpha was calculated to check the internal consistency and a value of 0.8 or above was deemed acceptable [29]. For convergent validity, a moderate association between OB and quality of life was hypothesized, as reported in previous research [2, 3, 5]. Spearman’s correlation coefficient was calculated for associations between OBQ11-A total score and the single item scores on overall perception of quality of life and overall perception of health, and each of the four domains of WHOQOL-BREF. A two-sided pvalue of 0.05 was considered significant for all statistical tests.

## Results

A total of 103 participants returned the data-collection instruments. Less than 2% of the missing values were found for a few items in OBQ11-A, and those cases were excluded from the analysis. The same was observed for all items on the WHOQOL, except for one item on satisfaction with sex life, which was deemed sensitive by participants and had a missing value among 16.5% of participants. Missing values for WHOQOL were handled based on the procedures outlined in the user manual [31]. The domain mean was substituted for cases in which up to two items were missing for the physical, psychological, and environmental domains. For the social relationship domain, the mean of the other items was substituted only if one item was missing. Participants with more than 20% of missing data were excluded from the analysis. Based on these procedures, one participant was excluded from the analysis involving the WHOQOL-BREF. Only 77 participants answered the content validity questions.

### Participants

The mean age of the participants was 36.58 (SD = 7.93, minimum = 24, maximum = 60), and 72% were female. Other demographic characteristics of the participants are shown in Table 1.

**Table 1.** Participant characteristics.

Demographic variables	N (103)	Percentage
Gender		
Male	43	41.7
Female	60	58.3
Nationality		
Qatari	13	12.6
Non-Qatari	90	87.4
Education		
High school	10	9.7
Diploma	13	12.6
Undergraduate	65	63.1
Postgraduate	13	12.6
Doctoral	2	1.9
Marital status		
Unmarried	22	21.4
Married	76	73.8
Divorced	5	4.9
Job		
Physician	3	2.9
Nurse	10	9.7
Allied Health	53	51.5
Admin	17	16.5
Others	19	18.4
Number of children		
No of children	33	32.0
One	9	8.7
Two to three	45	43.7
More than 3	14	13.6

### Test–retest reliability

Test–retest reliability was evaluated using OBQ11-A responses administered within an interval of 7–10 days. The setting and method of administration (self-administered) were the same at both time points, and participants did not report any significant life events during this interval. The mean OBQ11-A score at first administration was 17.17 (SD = 4.86) and at second administration was 17.19 (SD = 4.89). The ICC values for the total score was 0.928 and ranged from 0.724 to 0.866 for the individual items. The ICC values for all items of OBQ11-A are shown in Table 5, along with the 95% confidence intervals.

### Content validity

Most participants considered OBQ11-A to be relevant, comprehensive, and comprehensible. The majority (85.7%) of the respondents mentioned that they would recommend using OBQ11-A as a tool to measure OB, demonstrating its relevance. Separately, 83.1% of respondents felt that the questionnaire covered all aspects of OB, supporting its comprehensiveness. Regarding the comprehensibility of OBQ11-A, 87% of the participants felt that the questions were clear and easy. The percentages of agreement reported by participants for the questions on relevance, comprehensiveness, and comprehensibility are displayed in Table 2.

**Table 2.** Percentage of agreement in content validity survey (*n* = 77).

Content validity items	Question used	Agree *n* (%)	Undecided *n* (%)	Disagree *n* (%)
Relevance	Q3 – “recommend this questionnaire to measure occupational balance”	66 (85.7)	9 (11.7)	2 (2.6)
Comprehensiveness	Q2 – “covered all aspects of occupational balance”	64 (83.1)	11 (14.3)	2 (2.6)
	Q7 – “lacks important questions about occupational balance”	23 (29.9)	14 (18.2)	40 (52)
Comprehensibility	Q1 – “questions were clear and easy”	67 (87)	8 (10.4)	2 (2.6)
	Q4 – “took a lot of time and effort to fill in the questionnaire”	17 (22.1)	8 (10.4)	52 (67.6)
	Q5 – “questions appear to encourage a specific answer”	3 (3.9)	7 (9.1)	67 (87.1)
	Q6 – “difficult to answer the questions”	24 (31.2)	7 (9.1)	46 (59.8)

### Construct validity

#### Exploratory factor analysis

The Kaiser–Meyer–Olkin (KMO) value of 0.906 and Bartlett’s test of sphericity (*p* < .001) indicated the suitability of the factor analysis to be conducted. The results of the factor analysis revealed that one factor explained 49.94% of the total variance in the model. The factor loadings for each item of OBQ11-A are shown in Table 3.

**Table 3.** Factor loadings of the arabic occupational balance questionnaire (OBQ11-a).

Item content	Item	Factor
Balance between energy-giving/energy-taking activities	10	0.795
Satisfaction with the number of activities during a regular week	8	0.740
Have sufficient time for doing obligatory occupations	5	0.739
Satisfaction with how time is spent in everyday life	7	0.732
Balance between work, home, family, leisure, rest, and sleep	4	0.718
Balance between physical, social, mental, and restful occupations	6	0.717
Balance between obligatory/voluntary occupations	9	0.712
Balance between doing things for others/for oneself	2	0.711
Satisfaction with time spent in rest, recovery, and sleep	11	0.701
Time for doing things wanted	3	0.616
Having sufficient things to do during a regular week	1	0.562

### Internal consistency

Cronbach’s alpha for the OBQ11-A total score was 0.899, which was within the acceptable range. The results are shown in Table 4.

**Table 4.** Internal consistency results of arabic occupational balance questionnaire (OBQ11-a).

Item	Min–Max	Median	Corrected item-total correlation	Cronbach’s Alpha if item deleted
Item 1	0–3	2	.483	.897
Item 2	0–3	2	.642	.889
Item 3	1–3	2	.535	.895
Item 4	0–3	2	.649	.889
Item 5	0–3	1	.664	.888
Item 6	0–3	1	.645	.889
Item 7	0–3	1	.660	.888
Item 8	0–3	2	.668	.887
Item 9	0–3	1	.638	.889
Item 10	0–3	1	.730	.883
Item 11	0–3	1	.622	.890

**Table 5.** Intraclass correlation coefficients (ICC) and 95% confidence intervals (CI) for each item of the arabic occupational balance questionnaire (OBQ11-a).

		95% CI	
Item	ICC	lower	upper
Item 1	.749	.629	.830
Item 2	.851	.780	.899
Item 3	.866	.802	.909
Item 4	.796	.698	.862
Item 5	.724	.593	.813
Item 6	.779	.672	.850
Item 7	.827	.745	.883
Item 8	.860	.793	.905
Item 9	.819	.733	.878
Item 10	.813	.724	.874
Item 11	.774	.666	.847
Total score	.928	.894	.952

### Convergent validity

Statistically significant moderate correlations were found between the OBQ11-A total score and the single item on quality of life, *M* = 3.93 (*n* = 101; ρ = .31, *p* = .002); single-item on health, *M* = 3.76 (*n* = 101; ρ = .40, *p* < .001); physical health domain, *M* = 65.69 (*n* = 101; ρ = .52, *p* < .001); psychological health domain, *M* = 65.20 (*n* = 101; ρ = .49, *p* < .001), social relationships domain *M* = 67.25 (*n* = 101; ρ = .36, *p* < .001); and environment domain *M* = 64.06 (*n* = 101; ρ = .57, *p* < .001) in the WHOQOL-BREF.

## Discussion

This study examined the psychometric properties of OBQ11-A, particularly its test-retest reliability, participant-level content validity, construct validity, internal consistency, and convergent validity among healthy adults. Availability of validated OB measurement instruments could encourage rehabilitation professionals to consider OB as a focus and outcome of rehabilitation interventions and promote OB-related research.

Although preliminary validity for OBQ11-A was established in previous research, its test-retest reliability was not examined. This study confirmed the test-retest reliability of OBQ11-A. The results, ICC value of 0.928 for the total score is comparable to the original version of the questionnaire in Swedish [21]. The ICC values for each of the individual items were more than 0.7 which is the acceptable limit for scales used in research [29]. However, the lower bound of the 95% confidence interval was below 0.70 for items one, four, five, six and eleven. Lower ICC values for individual items were reported in the Spanish version of the OBQ as well [13]. Therefore, it is recommended to use OBQ11 total scores in research rather than analysing individual items. As for other translated versions of OBQ/OBQ11, only ICC values for the total scores were reported [5, 8, 13, 23, 26].

In this study, we used a survey method to examine the relevance, comprehensiveness, and comprehensibility of the OBQ11-A from the perspective of the participants. Such participant-level validity was not reported in previous studies of OBQ11. It has to be noted that 29.1% of participants felt that the questionnaire lacked important aspects of the OB, and another 18.2% were undecided. These participants might have felt that some aspects of OB specific to their culture were missing in the OBQ11-A. For example, findings from a study among occupational therapists in Iran suggested that cultural belonging was central to the participants, and doing occupations that meet community expectations also contributes to the perception of OB [33], which is not represented in OBQ11. Further research to explore specific cultural determinants in the perception of OB, if any, that are specific to Arabic culture could inform further modifications to OBQ11-A. Several authors have suggested that subjective assessments of OB alone do not suffice to understand the OB of a person [33–35] and have stated the need to employ multiple assessment methods [21] before any recommendations can be made to improve an individual’s OB. Separately, 31.2% of participants felt that it was difficult to answer the questions and 22.1% felt that it took a lot of time and effort for them to fill in the questionnaire, with another 10% of participants undecided for these two questions. These difficulties in responding to certain items relate to the difficulty in reflecting and judging one’s own balance in relation to one’s daily occupation [18]. Difficulties in discerning the terms “balance,” “sufficient,” and “variety” in relation to occupations [5] and uncertainties about “occupations one must do” [25] have been reported in other language translation studies. These are inherent to the concept of OB, and a brief description with examples to describe OB along with the questionnaire might lessen the ambiguities participants might face while filling out the OBQ11. Nevertheless, the OBQ11-A was shown to have acceptable content validity in terms of relevance, comprehensiveness, and comprehensibility for its use in research. In clinical settings, OBQ11-A could be used as a screening tool, and further interventions could be based on a dialogue with the clients about the rationale behind their rating on each of the items, their individual perspectives of balance, and that of community expectations [34].

Exploratory factor analysis was used to demonstrate the construct validity of OBQ11-A in line with previous studies [22, 26]. Nine of the 11 OBQ11-A items had factor loadings above 0.7, which is considered excellent [36]. Item one (having sufficient things to do in a week) had the lowest factor loadings, which is consistent with the findings from previous studies [22, 26]. All participants in the study were employed and probably occupied throughout the day, which could have made this item less relevant to their perception of OB [26].

Internal consistency of the Spanish, Swedish, Arabic, French, and Turkish versions of the OBQ has been examined in previous studies and was found to be acceptable to good [5, 13, 20, 22, 23, 26]. The findings from this study on the the internal consistency of OBQ11-A was consistent with the findings from other translated versions.

In previous studies, various health-related constructs have been used to examine the convergent validity of OBQ/OBQ11 including satisfaction with life [5, 13], life balance [23], psychological wellbeing [6], perceived health [8], and perceived stress [8] and all studies have reported a moderate positive association with OBQ/OBQ11 total scores. This study used quality of life to examine convergent validity of OBQ11-A and found a moderate association between OBQ11-A scores and the different domains of the WHOQOL-BREF questionnaire, supporting its construct validity. The highest associations (ρ > .5) were with the physical and environmental domains that covered pain and discomfort, energy and fatigue, sleep and rest, physical safety and security, home environment, financial resources, health and social care: accessibility and quality, opportunities for acquiring new information and skills, participation in and opportunities for recreation/leisure activities, physical environment (pollution/noise/traffic/climate), and transport [31]. The perception of OB is a reflection of an individual’s satisfaction with number and variety of occupations in daily life and the relationship between OB and the physical states and environmental affordances is understandable as these factors affect the ability of an individual to participate in different occupations. This relationship could manifest more for people undergoing rehabilitation which could be explored in future research.

### Limitations

Participants were limited to healthy employed adults, who were staff and visitors in two hospitals, which limits the validity of OBQ11-A to other population groups. It is recommended to report validity findings in future studies utilising OBQ11-A for other population groups. Prior knowledge about the concept of OB, which might have influenced the participants’ responses to the content validity questions, was not explored, which is another limitation. However, this is the first attempt to understand the participant-level content validity of OBQ/OBQ11, which is the strength of this study.

## Conclusion

OBQ11-A has acceptable psychometric properties for use in research on healthy populations. OB research is predominantly found in the Western world, and the validated OBQ11-A could stimulate OB-related research among Arab countries. Future research could explore and incorporate cultural elements of OB into OBQ11-A and its revisions to address cross-cultural validity and reliability for clinical use. However, the OBQ11-A could be used alongside other assessment methods to address OB issues in clinical rehabilitation settings.

## Data Availability

Data not available due to institutional restrictions.
